# Economic evaluation of patient navigation programs in colorectal cancer care, a systematic review

**DOI:** 10.1186/s13561-018-0196-4

**Published:** 2018-06-14

**Authors:** Chloé Gervès-Pinquié, Anne Girault, Serena Phillips, Sarah Raskin, Mandi Pratt-Chapman

**Affiliations:** 1 Research Institute for Environmental and Occupational Health (Irset-Inserm UMR1085), Ester Team – UFR Santé – Département de Médecine, Rue Haute de Reculée, 49045 ANGERS Cedex01, France; 2 EA MOS 7348 - French School of Public Health, 20 avenue George Sand, 93200 Saint Denis, France; 3 Institute for Patient-Centered Initiatives and Health Equity at the George Washington University Cancer Center, 2600 Virginia Avenue NW, Suite 300, Washington, DC 20037 USA; 4 L. Douglas Wilder School of Government and Public Affairs, Virginia Commonwealth University, Scherer Hall, #313923 W. Franklin Street, Richmond, VA 23284 USA

**Keywords:** Colorectal cancer, Patient navigation, Cost-benefit analysis, Health care costs

## Abstract

Patient navigation has expanded as a promising approach to improve cancer care coordination and patient adherence. This paper addresses the need to identify the evidence on the economic impact of patient navigation in colorectal cancer, following the Health Economic Evaluation Publication Guidelines. Articles indexed in Medline, Cochrane, CINAHL, and Web of Science between January 2000 and March 2017 were analyzed. We conducted a systematic review of the literature using Preferred Reporting Items for Systematic Reviews and Meta-Analyses (PRISMA) guidelines. The quality assessment of the included studies was based on the Consolidated Health Economic Evaluation Reporting Standards (CHEERS) checklist. Inclusion criteria indicated that the paper’s subject had to explicitly address patient navigation in colorectal cancer and the study had to be an economic evaluation. The search yielded 243 papers, 9 of which were finally included within this review. Seven out of the nine studies included met standards for high-quality based on CHEERS criteria. Eight concluded that patient navigation programs were unequivocally cost-effective for the health outcomes of interest. Six studies were cost-effectiveness analyses. All studies computed the direct costs of the program, which were defined *a minima* as the program costs. Eight of the reviewed studies adopted the healthcare system perspective. Direct medical costs were usually divided into outpatient and inpatient visits, tests, and diagnostics. Effectiveness outcomes were mainly assessed through screening adherence, quality of life and time to diagnostic resolution. Given these outcomes, more economic research is needed for patient navigation during cancer treatment and survivorship as well as for patient navigation for other cancer types so that decision makers better understand costs and benefits for heterogeneous patient navigation programs.

**Electronic supplementary material:**

The online version of this article (10.1186/s13561-018-0196-4) contains supplementary material, which is available to authorized users.

## Introduction

The impacts of cancer on individuals, caregivers, society and health care systems are profound. The National Cancer Institute estimates that in 2016, 1.6 million people in the United States will be diagnosed with cancer and nearly 600,000 will die from the disease [1]. Globally, over eight million lives lost and almost 200 million disability-adjusted life years were attributed to cancer in 2013 [2]. Close to $125 billion was spent on cancer care in the U.S. in 2010 [1], a figure anticipated to reach $173 billion by 2020 [3]. The growing cost of cancer care reflects successes in the field, such as increases in both the percentage of people who survive cancer and the number of years survived, with resultant costs of specialized care needs [4]. It also reflects failures: for example, inadequate coordination of care through an “increasingly specialized and fragmented health care system” [5], which can lead to service duplications, lower treatment adherence, poorer care quality, worse health outcomes, and increased costs for patients and payers [6–8].

Cancer cost must be considered in the context of health and health care disparities. Racial/ethnic minorities, low-income populations, and others from historically marginalized backgrounds tend to be diagnosed at later stages of disease progression, receive lower quality care and bear a disproportionate burden of disease. Racial/ethnic disparities in cancer cost an estimated annual $193 billion in premature death and $471.5 million in lost productivity in the United States alone [9]. As others have observed, there are both economic and moral arguments for bending the cost curve of cancer care [10]. Patient navigation (PN) has rapidly expanded as a promising approach to address cancer disparities, reduce the overall cost of cancer, and improve care coordination and patient adherence across the care continuum, particularly among minority and/or economically disadvantaged patient populations [11, 12].

PN programs have been effective in improving clinical outcomes and patient experience, including reducing patient distress and anxiety, shortening acute hospital stays, increasing patient satisfaction and empowerment, and reducing disparities in timely movement through the cancer care trajectory [13]. Yet PN’s effects on the cost of cancer care are not as well documented. Few studies on PN programs provide an exhaustive economic evaluation of their outcomes, and even fewer base their evaluation on validated methodological guidelines like the Consolidated Health Economic Evaluation Reporting Standards (CHEERS) and on well-defined coordination problems [14]. More rigorous economic analyses of PN are needed for a variety of reasons, not least to inform policy decisions about if and how to pay for PN services, which in the U.S. are currently not reimbursed by third party payers.

Strengthening understanding of the economic impacts of PN is particularly valuable for cancer types in which population-level early detection is cost-effective and PN improves adherence to initial phases of care. Colorectal cancer (CRC) is the third most commonly diagnosed cancer in the United States and worldwide. CRC will be diagnosed in an estimated 96,000 people in the United States in 2017 and will take the lives of over 50,000 people, disproportionately affecting racial/ethnic minorities and economically disadvantaged people due to later-stage diagnosis and low screening adherence [15]. Globally, almost 694,000 lives were lost to CRC in 2012 [2]; it is estimated that worldwide CRC diagnoses will nearly double in the next two decades to reach 2.4 million cases in 2035. The United States will spend approximately $17.41 billion on CRC care in 2020, with over half of cost spent on continuing care and in the last year of life [3]. Yet, the majority of spending on treatment, as well as the estimated $4.2 billion in productivity lost to CRC deaths and inestimable individual and family suffering [16], is largely considered avoidable due to the success of screening and removal of pre-cancerous polyps.

PN has demonstrated improvements in timely movement through the CRC care trajectory, particularly among racial/ethnic minority, low-income, and other disadvantaged populations [17]. Accordingly, it makes an excellent case study to examine the economic impacts of PN on care.

Our study aimed to analyze the literature and assess the level of evidence on the economic evaluation of PN programs in CRC.

## Methods

### Review process

A systematic search of the scientific literature was conducted in four major databases (MEDLINE using PubMed, Web of Science, Cochrane and CINAHL) to identify relevant English-language publications relating to economic evaluations of PN programs in CRC. The Preferred Reporting Items for Systematic Review and Meta-Analysis (PRISMA) guidelines were used to ensure systematic selection of studies [18] (see Additional file 1: Table A).

The three preconditions for inclusion were that the study:

1. evaluated PN services: we confirmed that each article explicitly addressed PN (including navigators with and without a clinical license such as nurses and social workers performing navigator functions) rather than other health care provider roles that may perform similar tasks,
2. conducted an economic evaluation, and
3. focused on CRC.

Keywords were defined according to population, intervention/comparator, outcomes, study design elements (see Additional file 1: Table B). Keywords were searched in the title or abstract of full-length publications that were published between January 2000 and march 2017.

Articles were excluded if they did not correspond to the above criteria. Systematic literature reviews were also excluded

### Study selection

Our initial search resulted in 243 articles that met the above-mentioned criteria. The retrieved studies were reviewed by four researchers in close consultation with a senior author (MPC) and, in case of disagreement, issues were resolved by consensus.

Duplicates were removed, resulting in a total of 121 articles for review. The 121 citations were screened on the basis of titles and abstracts. 16 papers were then selected.

The full-text articles for the 16 abstracts selected for inclusion were retrieved and read. The final number of original empirical studies was 9 after assessment of eligibility for inclusion.

Data was extracted independently by four researchers. Extracted information included: bibliographic details, information on participants, PN interventions, outcomes, study design, and results. Disagreements were resolved by discussion.

**Fig. 1:**
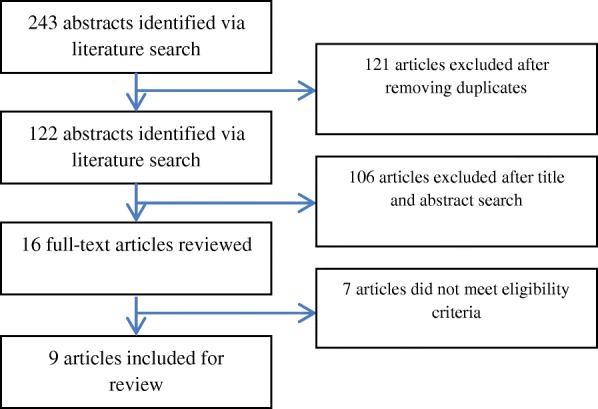
Search flow

Figure 1 provides a PRISMA diagram illustrating details of the search strategy.

### Quality assessment

The studies identified for inclusion were assessed against the 24 key criteria contained in the CHEERS checklist [19]. The checklist has been jointly endorsed by ten journals. All items were presented in the tables for this review, consisting of five broad categories: Title and abstract (2 items); Introduction (1 item); Methods (14 items); Results (4 items) and Discussion (3 items) (see Additional file 1: Table C).

In certain studies, we considered that some CHEERS’ items were not applicable:

- When the time horizon was less than one year, discounting (item 9) was considered not applicable
- When the economic evaluation was a cost analysis, effectiveness measurement (item 11) was considered not applicable
- When measured outcomes were not preference-based, preference measurement (item 12) was considered not applicable.

We used the results of the quality assessment for descriptive purposes and to investigate potential sources of heterogeneity.

### Cost classification used

The costs considered were:

- Direct costs encompass all the health care expenditures generated by the program. They include the resources used for program implementation (program cost) and both the medical and non-medical resources generated as a consequence of the program (e.g., physician consultations, treatment cost, professional home care). These resources are priced on the health care market (consultation cost, treatment cost, etc.).
- Indirect costs correspond to resources without a market price, such as opportunity costs for both the patient (e.g., travel time, waiting time, and productivity loss on the labor market) and his/her relatives (since informal care time means the caregiver cannot pursue other activities). While necessarily estimated, these resources are given a monetary value to be integrated within the costs of the economic evaluation.

## Results

We present in Additional file 1: Table C the quality assessment of the included studies based on the CHEERS checklist. It shows that seven out of the nine studies reviewed can be considered high quality studies, following an existing approach to determining quality in cancer scholarship [20], with an average proportion of 84.8% of checklist criteria fulfilled.

Table 1 shows the main characteristics of the studies included [21–29]. Most articles (*n* = 6) exclusively addressed navigating those due for recommended CRC screening to receive those services (*n* = 6). The few articles examining PN to diagnostic resolution (*n* = 2) addressed multiple cancer types. Two studies compared PN to screening colonoscopy versus other screening modalities (fecal occult blood testing (FOBT) or fecal immunochemical testing (FIT)). All studies but one occurred in the United States and took place in various clinical settings, primarily in the health care safety net setting. At least two-thirds of studies focused on racial/ethnic minority, low-income, or otherwise underserved populations. The only study to address PN from confirmed diagnosis through treatment or end of life occurred in New Zealand.

**Table 1:** General characteristics of the studies included

Authors	Target population	Cancer type and navigation continuum phase	Navigator profile	Study design	Time horizon
Donaldson (2012)	959 breast cancer and 411 colorectal cancer patients; African American, White, Hispanic and other race; Low-income/ underserved populations.	Breast, Colorectal Screening to diagnostic resolution	Characteristics: lay patient navigator (Washington, DC); non-clinically licensed patient navigator (Kentucky); non-clinically licensed patient navigator, nurse-LPN, bilingual outreach worker (Louisiana) Tasks: Unspecified	2 arms among 3 community hospitals: comparison between usual care (1), PN program (2)	12 months
Elkin (2012)	25,481 low-income, high risk, urban, majority Hispanic	Colorectal To Screening	Characteristics: Lay health educators Tasks: 1. appointment reminders; 2. colonoscopy and bowel preparation education; 3. management of patient concerns; 4. referral to financial services	Quasi-experimental with pre-post; 2 arms (3 intervention and 3 comparison hospitals); usual care (1), PN program (2)	Pre-program period: 12 months Post-program period: 1 month
Jandorf (2013)	Socioeconomically disadvantaged 700 patients referred for colonoscopy by primary care providers between May 2008–May 2010, age 50+, due for colorectal screening (colonoscopy > 5 years, not up to date with other forms of screening)	Colorectal To Screening	Health worker Characteristics: 3 types of navigators were used due to objectives for a different study using same patient population: 1. Professional navigators with formal health education training, African American race concordant; 2. Peer navigators (“lay” individuals > 50 years old from East Harlem who had undergone colonoscopy) with study-provided training, African American race concordant; 3. Professional navigators, language concordant but not necessarily race concordant for non-African American patients Tasks: 1. Appointment scheduling; 2. Patient education on colonoscopy procedure and preparation; 3. Appointment reminder calls (multiple); 4. Transportation needs assessment; 5. Confirmation that mailed information was received; 6. Concerns addressed; 7. Depending on study arm, script about importance of colorectal cancer screening, discussion of navigator’s colonoscopy experience, and/or impact of colorectal cancer on African Americans.	RCT Single-arm (4 navigation arms for separate RCT study were aggregated and not compared in this study)	24 months
Bensink (2014)	10,521 patients with breast, prostate, colorectal or cervical cancer screening abnormalities (5063 navigated, 5458 usual care). Mostly minority (39% Hispanic, 32% African American), 40% publically insured adult population.	Breast, Prostate, Colorectal, Cervical Screening to diagnostic resolution	Characteristics: Professional health workers and/or lay persons Tasks: 1. “support and guidance for timely access to the cancer care system,” 2. “addressing barriers,” 3. “facilitating quality care.”	Research designs varied among sites: Individually RCT (2 sites); Group RCT (2 sites); Quasi-experimental (5 sites)	12 months
Ladabaum (2015)	Hypothetical cohort of 10,000 adults (43% African American, 49% Hispanic, 4% white, 4% other) entered into model at age 50 and followed until age 100 or death	Colorectal To Screening	Characteristics: Unspecified Tasks: Unspecified	Comparison between (1) usual care (2) PN program (3) Fecal occult blood test or fecal immunochemical test.	Life time
Lairson (2014)	945 patients age 50–79 noncompliant with USPSTF colorectal cancer screening recommendations, with visit at participating practice in the last 2 years), mostly White	Colorectal To Screening	Characteristics: Nurse navigator Tasks: 1. Structured follow up call to confirm receipt of mailed CRC screening materials and to answer questions; 2. Reassessment of screening preference; 3. Encourage screening completion; 4. Provide instructions for stool blood test or identify colonoscopy locations (no appointment scheduling); 5. Provision of additional information if screening preference changed.	RCT, pre-post, 3 arms (usual care; mailed information and referral phone number; mailed information and nurse navigator follow up)	12 months
Blakely (2015)	Stage III colon cancer patients	Colorectal Diagnosis through Treatment and End of Life	Characteristics: hospital-based clinical nurse specialist Tasks: 1. “providing information and support for the patient,” 2. “identifying and addressing patient barriers to accessing care (transport/ financial/ social)”, 3. “coordinating arrangements for pre-operative assessments and hospital admission,” 4. “optimising post-operative care,” 5. “Tracking investigations and appointments,” 6. “ensuring the patient is discussed at a multidisciplinary team meeting,” 7. “Making referrals as necessary,” 8. “acting on any administrative delays.”	Comparison between (1) Usual care; (2) PN program.	Lifetime after diagnosis
Meenan (2015)	Patients due for colorectal screening (colonoscopy > 9 years, sigmoidoscopy > 4 years, fecal occult blood test > 9 months)	Colorectal To Screening	Characteristics: 2 part-time nurse navigators (combined 10% full-time equivalent weekly) Tasks: 1. assistance with colorectal screening decision-making; 2. follow up on fecal occult blood tests with no laboratory results after 3 weeks; 3. assessment of colorectal cancer risk; 4. review of procedural risk; 5. motivational counseling to define patient screening intent; 6. creation of patient-shared screening action plan; 7. referrals assistance; 8. appointment assistance; 9. endoscopy preparation assistance; 10. test completion tracking.	RCT, 4 arms: (1) usual care; (2) automated electronic health record-linked mailings; (3) automated mailing with telephone assistance; (4) automated mailing, telephone assistance, and nurse navigation services	24 months
Wilson (2015)	461 Hispanic men, low-income, uninsured, 50+ years old, member of Bexar County’s financial assistance program, with no colorectal cancer screening in the last 10 years.	Colorectal To Screening	Characteristics: Bilingual community health worker, Bilingual program coordinator Tasks: 1. colorectal cancer and colonoscopy patient education; 2. discussions on colorectal cancer with immediate family; 3. liaison between Hispanic communities and patient care services; 4. encouragement of colonoscopy appointment scheduling; 5. addressing questions and concerns throughout process, 6. home visits as needed, 7.transportation assistance, 8. Social support, 9. Appointment coordination and scheduling, 10. Setup of appointment reminders.	Comparison between (1) Usual care, (2) PN program.	24 months

Navigator profiles and roles described in the articles were diverse. Three studies used nurse navigators; four used non-clinically licensed navigators with various titles such as “lay” health educator or outreach worker. One article included a licensed clinical social worker and at least two employed bilingual staff. For the seven studies that described navigator actions, navigators provided assistance through a wide range of tasks. These included identification and removal of barriers to care, coordination of appointments and referrals, appointment reminders, support and encouragement, information and education, and tracking and follow up. Among the reviewed studies, four were based on randomized controlled trials (RCTs). All the studies reviewed indicated the time horizon for evaluation.

Table 2 shows that most of the studies (*n* = 8) adopted the health care system perspective, which refers to a variety of entities including the hospital (*n* = 3) or public or private payers (*n* = 5). Six studies were presented as cost-effectiveness analyses (among which, one presented both a cost-effectiveness analysis and a cost-benefit analysis), one was presented as a cost-utility analysis, one was a cost-consequence analysis, and one was a cost analysis. If we assume that using Quality Adjusted Life years (QALYs) implies conducting a cost-utility analysis [30], two of the cost-effectiveness analysis reviewed were also cost-utility analyses.

**Table 2:** Key findings on the economic effects of PN programs

Authors	Economic impact	Economic outcome	WTP (preference measurement)	Choice of health outcomes	Study perspective	Type of economic evaluation	Model and Estimating resources and cost	Direct costs considered	Indirect costs considered
Donaldson (2012)	PN cost-effective	ICER was $3567 per diagnostic resolution (range $1192 to $9708 depending on the model assumptions).	Unspecified	Time from abnormal finding to diagnostic resolution; Loss to follow-up after an abnormal finding	Health care system (payer)	Cost-effectiveness analysis	Decision analytic model; Model-based economic evaluation Data sources: Scientific literature; published sources from several health maintenance organizations in USA.	Program costs: Personnel, travel, phone/communication charges, office supplies, training Medical costs: Treatment cost including additional care provided	None
Elkin (2012)	PN cost-effective and financial benefit	ICER varied from $199 to $708 per additional colonoscopy (depending on the context)	Unspecified	Receipt of colonoscopy	Health care system (provider)	Cost-effectiveness and cost-benefit analyses	Decision analytic model; Model-based economic evaluation Data sources: NYC Department of Health and Mental Hygiene and Health and Hospitals Corporation records, Medicare reimbursement rates	Program costs: Personnel, phone/communication charges Medical costs: colonoscopy	None
Jandorf (2013)	PN generates additional income	Current PN model was $35,035.50 more profitable than historical PN model and $44,956more profitable than the national average	Unspecified	% of complete screening colonoscopy (fixed ex-ante for each intervention considered)	Health care system (provider)	Cost-analysis	No decision analytic model Data sources: Mount Sinai’s business office; National Health Interview Survey (NHIS) as	Program cost: personnel (salaries of the Pro-PNs) and supplies (printed materials mailed to participants, paper, and postage costs), add on costs (bowel preparation, car service Medical cost: colonoscopy procedure (patient costs, support services)	None
Bensink (2014)	PN borderline cost- effective	The total adjusted incremental cost of navigation vs. usual care was $275 (95% CI: $260 to $ 290)	Unspecified	Time from abnormal finding to diagnostic resolution	Societal	Cost-consequence analysis	No decision analytic model stated. Data sources: PNRP study records; Medicare fee schedules published by the Centers for Medicare and Medicaid Services	Program costs: Overhead, office equipment, personnel, travel, phone/communication charges, office supplies, training, staff recruitment Medical costs: Diagnostic follow-up tests and services	Travel cost; waiting time for medical care (patient)
Ladabaum (2014)	PN cost-effective	ICER was *$9800 per QALY gained compared with colonoscopy without navigation *$5300 per QALY gained compared with no screening *$23,800 per QALY gained compared with FOBT, 40% uptake *$26,000 per QALY gained compared with FIT, 40% uptake *$118,700 per QALY gained compared with FOBT, 65% uptake	Unspecified	QALY (screening uptake, number of cases of cancer, number of colorectal deaths)	Health care system (payer)	Cost-effectiveness analysis (cost-utility analysis)	Decision analytic model (Markov); Model-based economic evaluation Data sources: Cancer screening studies, 1992 SEER data, Medicare reimbursement rates, published sources from several health maintenance organizations in USA.	Program costs: Completer costs (not specified) Medical costs: Colonoscopy; sigmoidoscopy; adverse events, stage-specific cost of treatment	None
Lairson (2014)	PN cost-effective	*The ICER was $1958 (95% CI, $880–$9043).when we compared the standard intervention group with the TNI (tailored navigation intervention) group	For a $1200 WTP the probability of cost-effectiveness increases to 0.90 comparing the SI with usual care, and it increases to 0.56 comparing the TNI with the usual care. * For a $1200WTP the probability of cost-effectiveness of the TNI versus the SI is only 0.16 (within the highest cost scenario) * For a $1000WTP the probability of cost-effectiveness of the TNI versus the SI is only 0.11.	Receipt of colonoscopy	Health care system (provider)	Cost-effectiveness analysis	Decision analytic model; single-study based economic evaluation Study invoices; current market prices for supplies	Program costs: Overhead, personnel, phone/communication chargers, office supplies, training	None
Blakely (2015)	PN cost-effective	ICER of Was $ 15,600) per QALY gained compared to ‘business-as-usual’	PN program is cost-effective for a willingness to pay of $16,500 (using mean value) or $ 21,000 (using the upper uncertainty limit).	QALY -disability weight (reduction in delays, better adherence to chemotherapy)	Health care system (payer)	Cost-utility analysis	Decision analytic model (discrete event simulation model); Model-based economic evaluation Data sources: Scientific; New Zealand Cancer Registry data, Expert estimates; local health care Professionals; referrals	Program cost: Personnel, overhead Medical costs: consultation, chemotherapy, dietitian, social worker	None
Meenan (2015)	PN cost-effective	*$465 per additional screened individual, compared to automated arm *$496 per additional screened individual, compared to telephone assisted arm * $65 per additional screened individual, compared to usual care arm	*Above WTP values of approximately $500 for an additional screened person, navigated intervention is most likely to be cost-effective (40% probability of cost-effectiveness) * A $1697 WTP is associated with a 95% probability of navigated being cost-effective	Receipt of colonoscopy in the 2-year follow-up period	Health care system (payer)	Cost-effectiveness analysis	Decision analysis (Probabilistic – monte carlo –simulation); Single study-based economic evaluation Data sources: data collected for the trial.	Program costs: Personnel, phone/communication charges Medical costs: sigmoidoscopy, colonoscopy, blood tests	None
Wilson (2015)	PN cost-effective	ICER is estimated at $3765 per QALY gained	Unspecified	QALY; Life Years; Life expectancy	Health care system (payer)	Cost-effectiveness analysis (cost utility analysis)	Probabilistic simulation model (Markov); Model-based economic evaluation Data sources: Scientific literature; Navigation program records	Program costs: Personnel, travel, “other” Medical costs: Colonoscopy; polypectomy, cost of treatment including treatments for terminal care	None

All studies computed the direct costs of the program, which were defined a minima as the program costs, including training, personnel, and supply costs. Eight studies considered direct medical costs, which were usually divided into outpatient and inpatient visits, tests and diagnostics. Estimated treatment cost was only considered in four papers and no study included direct non-medical cost, such as home care expenses. Only one study included indirect costs in the total costs associated with the PN program, including patient productivity loss and travel cost. No study included indirect costs associated with informal care. The clinical outcomes studied were mainly measures of time from abnormal finding to diagnostic resolution (*n* = 2), receipt of colonoscopy (*n* = 4), Quality Adjusted Life Years (*n* = 3) or Life years (*n* = 1). One third of the studies interpreted their results in relation to different stakeholders’ willingness to pay (WTP) for improvements in care.

All but one study concluded that PN programs were unequivocally cost-effective for the health outcomes of interest. For instance, Incremental Cost Effectiveness Ratios (ICERs) ranged from $65 to $1958 per additional screening meaning that adopting the PN program instead of the alternative care strategy considered (for instance usual care, or fecal occult blood test or automated electronic health record-linked mailings) leads to a cost of $[65 to 1958] for an additional screened patient. ICERs ranged from $1192 to $9708 per diagnostic resolution and from $3765 to $15,600 per QALY gained. There was high probability for PN to be cost-effective for CRC if stakeholder’s WTP ranged between $1200 and $1697 per additional screening and from $16,500 to $21,000 per QALY gained. In comparison, the National Institute for Health and Clinical Excellence (NICE) has been using a cost-effectiveness threshold ranging between £20,000 and £30,000 ($27,000 – $40,000) – usually per QALY gained - for over 14 years [31, 32]. The remaining study concluded that PN programs were only likely to be cost-effective (at $43,520 per life-year saved) under the most favorable assumptions, in which patients lost to follow-up have more advanced cancer, and navigators account for a 6-month earlier time to diagnostic resolution and have a 15% higher probability of follow up resolution completion [24].

## Conclusions

Most PN programs for CRC presented in our review had high probability for being cost-effective compared to usual care, given a conservative cost-effectiveness threshold of $50,000 per QALY gained [33]; one study found one-time PN to be cost- saving. Cost-effectiveness evidence is most robust for PN programs designed to increase adherence with CRC screening using colonoscopy. Given the U.S. Preventive Services Task Force’s A grade recommendation of CRC screening and the demonstrated success of PN to increase screening adherence among racial/ethnic minorities, low-income populations, and other disadvantaged patients, the volume and strength of the evidence in favor of the economic value of PN for colorectal screening adherence is unsurprising.

There are fewer articles for other phases of the continuum or using other screening methods [34]. The scant evidence seems to be tentatively favorable for the phase from abnormal screening to diagnostic resolution. Donaldson et al. (2012) concluded that PN programs increased achievement of timely diagnostic resolution for CRC (as well as breast cancers) among largely uninsured patients, and would be cost-saving if they were able to avert three to four cancer deaths per year [21]. Bensink et al. (2014) found limited economic benefit for PN during this phase (across four cancer types), indicating the greatest cost-effectiveness for those with the greatest needs such as the longest lapses in follow-up after screening, the most severe screening results, or the greatest potential to make gains in timeliness [24]. Lairson et al. advised payers to consider covering the costs of patient navigation for colonoscopy, which, compared to FOBT, has more chance to be considered cost-effective and even cost-saving when adopting larger time horizons [25]. The only study examining PN during the treatment phase addressed stage III colon cancer patients. Blakely et al. found PN to have high probability of cost-effectiveness, even considering a conservative WTP threshold [26]. These findings provide initial promising evidence for decision makers in support of PN for patients with more advanced cancers, and also for PN roles in providing treatment coordination and support.

Evidence of PN’s cost-effectiveness is bolstered by the methodological soundness of the studies included in this review. Seven studies, all published after 2012, meet standards for high-quality based on CHEERS criteria. One of the studies was a cost analysis, making an incremental interpretation of the results impossible according to CHEERS guidelines [23].

Although there have been calls for establishing common PN cost measures [35], establishing such measures are challenging since costs to one stakeholder is revenue for another. In the studies we reviewed, there were variations in considerations and definitions of direct costs, indirect costs and health outcomes. The lion’s share of the total cost of PN programs was most often attributable to direct medical costs rather than direct non-medical costs or indirect costs not covered by health insurance [36]. In other words, most of the reviewed studies adopted the health care system perspective rather than society’s perspective.

Only a third of the articles addressed stakeholders’ WTP. WTP is an important consideration to help payers optimize resource allocation, in particular with PN programs that are more costly but also more effective [28]. The perspective adopted is also crucial to a discussion of WTP thresholds, especially since PN programs considered to be cost-effective for society may exceed a hospital administrator’s budget constraint, or their WTP, corresponding to their preferences for an improvement in patient’s health outcome thanks to PN. It is noteworthy that patient preferences and patient reported outcomes (PROs) associated with PN are not addressed in the studies reviewed.

While this review advances understanding of the cost-effectiveness of CRC PN, findings should be interpreted with caution given limitations to current extant research. The heterogeneity of PN programs impedes the generalizability and comparability of individual and aggregate findings. The diversity of navigator roles, modes of communication and intensity of the interventions not only have the potential to produce heterogeneity in PN outcomes; it also produces variation in direct costs related to personnel and program costs. In settings in which PN occurs within a multi-faceted approach isolating PN-specific outcomes from aggregate outcomes may be especially challenging [23, 35]. For instance, the intervention described by Wilson and Villarreal to increase colonoscopy adherence includes free colonoscopies, extended clinic hours, and taxi services [29]. Another problem affecting the generalizability of the results is the definition of usual care which was appreciably different across the studies reviewed. Further research could consist in comparing PN programs by navigator profile in addition to (or even instead of) being limited to a specific pathology. This kind of comparison would require detailed characteristics about navigator profile, such as their academic background, professional training, level of remuneration, length of work experience, etc. that are missing in most of the studies reviewed.

While PN program implementation is characterized by significant variability, the screening method and phases of the cancer continuum studied were limited among the studies examined. Therefore, these cost-effectiveness evaluation results may not apply for PN interventions with screening methods and at cancer continuum phases not included in this review. Extrapolation of findings for PN cost-effectiveness for other types of cancers should be done with extreme caution given that colonoscopy screening doubles as a preventive procedure, extending savings of early detection and removal of polyps over a lifetime. Colonoscopy is thus unique among cancer screening modalities.

Finally, our review faced several of the challenges often found in economic reviews. Economic modeling is complex. Multiple different models were used across the studies included in review, and results could have been affected by each model’s type, structure, data sources and assumptions [37]. Lack of cost-benefit analyses prevented us from assessing whether PN could be profitable for providers, health care systems and societies (and at what cost for payers and possibly patients), but such analyses could move scholarship beyond cost-effectiveness.

## Additional file

Additional file 1: Additional file of Economic evaluation of patient navigation programs in colorectal cancer care, a systematic review. (DOCX 38 kb)

## Abbreviations

CHEERS: Consolidated Health Economic Evaluation Reporting Standards
CRC: Colorectal cancer
FIT: fecal immunochemical testing
FOBT: fecal occult blood testing
ICER: Incremental Cost Effectiveness Ratio
PN: Patient navigation
PRISMA: Preferred Reporting Items for Systematic Review and Meta-Analysis
PRO: patient reported outcomes
QALY: Quality Adjusted Life years
RCT: randomized controlled trials
WTP: willingness to pay
